# Nutrition perceptions, needs and practices among patients with plasma cell disorders

**DOI:** 10.1038/s41408-022-00666-w

**Published:** 2022-04-20

**Authors:** Maria A. Malik, Nathan W. Sweeney, Mohammad Jafri, Andriy Derkach, Cynthia Chmielewski, Peter A. Adintori, Sham Mailankody, Neha Korde, Carlyn R. Tan, Hani Hassoun, Malin Hultcrantz, Jens Hillengass, Susan E. McCann, Neil Iyengar, Saad Usmani, Sergio A. Giralt, Ola Landgren, Marcel R. M. van den Brink, Jennifer M. Ahlstrom, Alexander M. Lesokhin, Anita D’Souza, Susan Chimonas, Urvi A. Shah

**Affiliations:** 1 Geisel School of Medicine at Dartmouth, Hanover, NH USA; 2 Myeloma Service, Department of Medicine, Memorial Sloan Kettering Cancer Center, New York, NY USA; 3 HealthTree Foundation, Lehi, UT USA; 4 Memorial Sloan Kettering Cancer Center, New York, NY USA; 5 Department of Biostatistics and Epidemiology, Memorial Sloan Kettering Cancer Center, New York, NY USA; 6 Department of Medicine, Adult Bone Marrow Transplant Service, Memorial Sloan Kettering Cancer Center, New York, NY USA; 7 Department of Medicine, Roswell Park Comprehensive Cancer Center, Buffalo, NY USA; 8 Department of Cancer Prevention and Control, Roswell Park Comprehensive Cancer Center, Buffalo, NY USA; 9 Department of Medicine, Breast Service, Memorial Sloan Kettering Cancer Center, New York, NY USA; 10 Cellular Therapeutics Center, Memorial Sloan Kettering Cancer Center, New York, NY USA; 11 Myeloma Program, Department of Medicine, University of Miami, Sylvester Comprehensive Cancer Center, Miami, FL USA; 12 Division of Hematology/Oncology, Department of Medicine, Medical College of Wisconsin, Milwaukee, WI USA; 13 Center for Health Policy and Outcomes, Memorial Sloan Kettering Cancer Center, New York, NY USA

**Keywords:** Cancer prevention, Myeloma

## Introduction

As patients with Plasma Cell Disorders (PCDs) live longer due to therapeutic advances, outcomes may be further improved by optimizing nutrition. Additionally, monoclonal gammopathy of undetermined significance (MGUS) and low- to intermediate-risk smoldering multiple myeloma (SMM) present unique opportunities for early intervention, given the standard of care is observation over time [1].

Epidemiologic studies have confirmed that diet is a known risk factor for PCDs [2]. Two large prospective cohort studies support that Western diets, noted for their high inflammatory or insulinemic potential, may be linked to an increased risk of multiple myeloma (MM), while vegetarians and vegans have decreased risk compared to meat-eaters [3, 4]. Further studies based on individual dietary components suggest that increased consumption of fruits, vegetables, whole grains, and seafood is associated with a reduced risk of PCDs [5–7] (https://pubmed.ncbi.nlm.nih.gov/9639389/). In addition, MM-specific mortality is lower in patients with healthful pre-diagnosis dietary patterns, suggesting the potential for diet to affect survival outcomes as well [8]. Although the exact mechanistic basis of diet in plasma cell dyscrasias is unknown, early studies suggest the microbiome may play a significant role (https://www.medrxiv.org/content/10.1101/2022.03.29.22272361v1).

Patients with PCDs are often interested in learning how to optimize their physical health through diet, but oncologists and hematologists commonly do not address these concerns possibly due to the lack of PCD-specific dietary guidelines, although general guidelines by the American Institute for Cancer Research (AICR) and the American Cancer Society (ACS) for cancer prevention and survivorship do exist [9, 10]. Therefore, they are applicable to MGUS and SMM in addition to MM. The aim of this 24-question online survey was to explore patients’ nutrition information needs, perceptions, and practices and to identify areas for further research.

## Subjects and methods

We utilized HealthTree® Cure Hub, an online tool created by HealthTree Foundation (a division of the 501(c)3 non-profit organization, CrowdCare Foundation), and invited participants with PCDs to answer questions pertaining to their diet and nutrition and related experience with their hematologists and oncologists [11]. This study was reviewed by the Memorial Sloan Kettering Cancer Center Institutional Review Board and determined to be exempt from further review (IRB X20-091). Over 8000 patients with a known history of PCDs in the United States had access to this survey from January to June 2021. Participants provided written informed consent at survey initiation. Deidentified survey responses and pre-collected health data for each participant were retrieved through the HealthTree platform at study conclusion. Summary statistics were used to estimate the distribution of responses across questions as a function of the number of participants that answered a given question. Differences in question responses between patients diagnosed with malignant (primary plasma cell leukemia (PCL), MM) versus precursor conditions (MGUS, SMM, plasmacytoma) were tested using Fisher’s exact test. McNemar’s Chi-square test was used to assess dietary shifts pre- and post-diagnosis.

## Results

We obtained 421 survey respondents: 205 (49%) ≤65 years, 153 (36%) male, and 282 (67%) white. A range of PCD diagnoses were represented, including 299 (71%) MM, 63 (15%) SMM, 18 (4%) MGUS, 6 (1%) solitary plasmacytoma, 1 (0%) PCL, and 34 (8%) unknown. There was no statistically significant difference in survey responses between those diagnosed with malignant versus precursor conditions. Overall, the majority of respondents (82%) reported having questions or concerns about diet and nutrition (i.e., foods to eat or avoid, portion sizes, and special diets) while fewer than half (43%) indicated that their hematologist or oncologist either appropriately addressed them directly (23%) or referred the patient to a dietician or nutritionist (20%). Moreover, 57% stated that diet and nutrition were not addressed by their hematologists or oncologists at all and 23% stated this topic was not addressed despite asking. Most patients (71%) reported that their hematologist or oncologist spent <10 min discussing nutrition with them; 41% spent 0 min (Table 1).

**Table 1:**
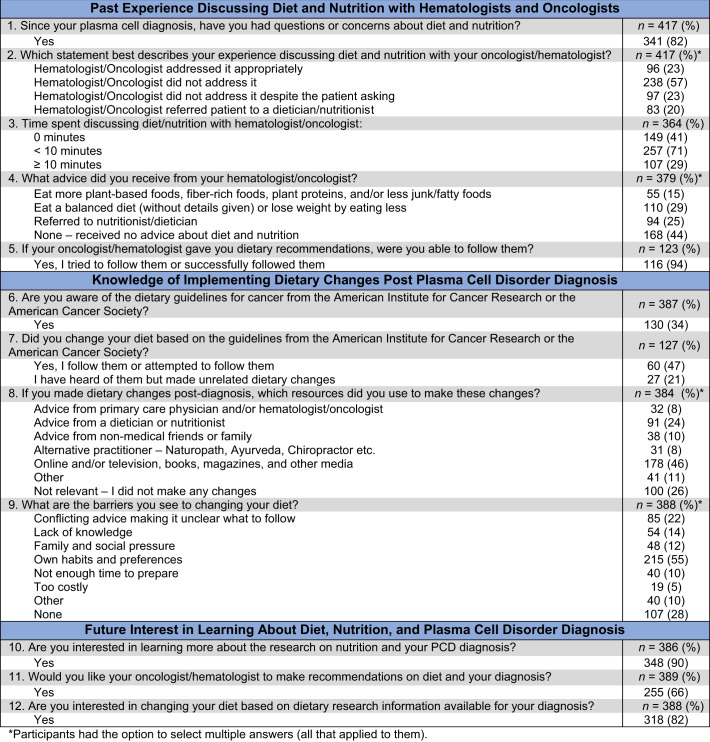
Perceptions and Experiences with Hematologists and Oncologists Regarding Diet and Nutrition.

About a third of respondents (29%) reported receiving non-specific dietary advice from their hematologist or oncologist, such as to eat a “balanced diet” or to consume less to lose weight, while 15% reported receiving more detailed meaningful guidance (i.e., recommended specific plant-based foods, fiber-rich foods, plant proteins, and/or less junk/fatty foods). Survey results reveal that of the patients that were able to receive dietary recommendations from hematologists or oncologists, the vast majority (94%) stated that they attempted to follow the advice. Additionally, although the ACS and the AICR have published dietary guidelines, 34% of respondents were aware of these guidelines, and of this group 47% attempted to follow them (Table 1).

Lack of knowledge and conflicting advice were barriers to making dietary changes for 14 and 23% of respondents, respectively. Presently, most receive post-diagnosis dietary guidance from non-medical sources, online, television, books, magazines, and other media (46%), advice from non-medical friends or family (10%) and alternative practitioners (naturopath, Ayurvedic doctor, chiropractor, etc.) (8%). Hematologists, oncologists, or primary care providers were a resource in making post-diagnosis dietary changes for 8%, and 24% received advice from dieticians or nutritionists (Table 1).

Most respondents (90%) indicated that they were interested in learning more about nutrition research and their diagnosis, 82% confirmed their interest in changing their diet based on this research, and 66% expressed that they would like their oncologist to make recommendations (Table 1). The most common motivating reasons reported by patients for implementing dietary changes include feeling better physically (68%), taking more control of one’s health (62%), feeling better emotionally (47%), looking better (42%), and losing weight (37%).

A significant number of patients self-reported that they consumed a healthier diet after diagnosis (75% pre versus 88% post, *p* < 0.0001). Furthermore, among patients with a self-reported unhealthy diet pre-diagnosis, 73% improved their diet post-diagnosis. In contrast, 6% with a healthy diet pre-diagnosis worsened their diet post-diagnosis (Table 2). Patients reported consuming food groups such as whole fruits, vegetables, whole grains, plant proteins, plant-based dairy, and seafood at significantly higher rates post-diagnosis (*p* < 0.0001). There was a concurrent decrease in the consumption of red meats, dairy products, sweetened drinks, and junk foods (Table 2).

**Table 2:**
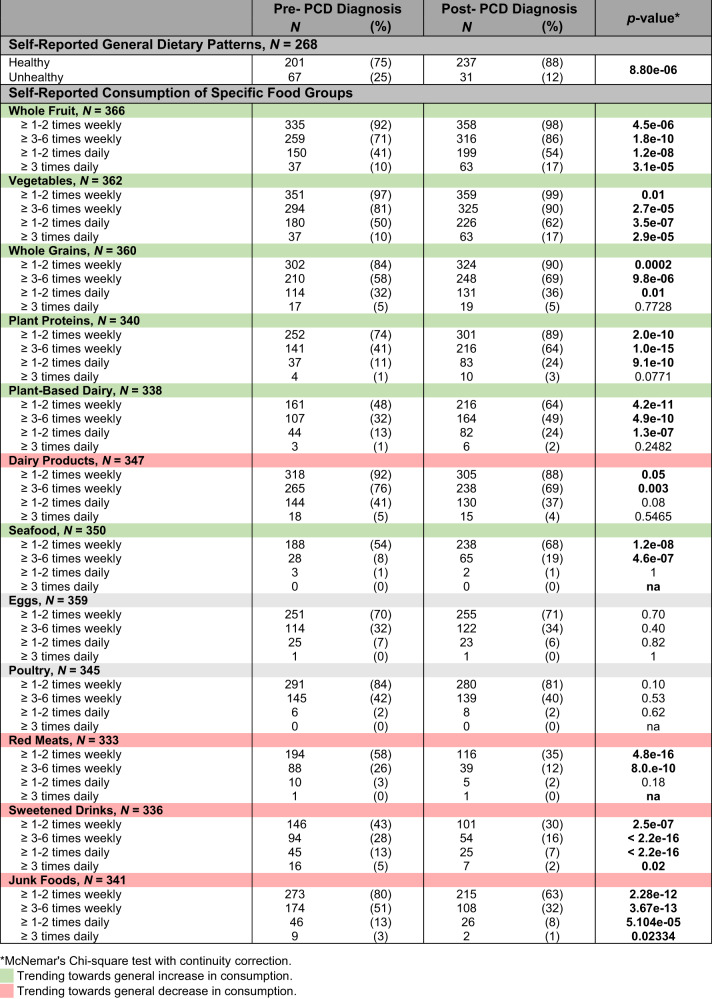
Self-reported dietary patterns in patients pre-PCD diagnosis versus post-PCD diagnosis.

## Discussion

Survey responses indicated that patients often change their diets post-diagnosis, suggesting that they may be amenable to dietary interventions. Cancer patients have been well-documented to make dietary changes following a diagnosis, and this trend extends to PCD patients [12] (https://pubmed.ncbi.nlm.nih.gov/12616253/). A cancer diagnosis can induce psychological stress which can motivate individuals to reduce known risk factors and promote general health [13]. Our results confirm that besides patients with active plasma cell malignancies, patients with precursor conditions such as MGUS may be similarly empowered to make dietary and lifestyle changes as they are apprehensive about their cancer progression risk. The lack of difference in survey responses between patients with active cancer and precursor disorders maybe due to the small sample size. The role of diet is possibly different across the plasma cell disorder spectrum and may be dependent on disease stage, nutritional status, comorbidities, and patient preference.

Additionally, a meta-analysis evaluating the effectiveness of primary care-based dietary interventions showed that personalized guidance from healthcare professionals can usher sustainable healthy diets in patients (https://doi.org/10.1002/1099-1611(200009/10)9:5%3C418::AID-PON474%3E3.0.CO;2-E). This suggests that patients who get professional guidance make healthier shifts. Of the 123 patients that reported receiving dietary advice directly from hematologists and oncologists, an overwhelming 94% stated that they attempted to follow the advice. This highlights the positive influence physicians may have in propelling healthful dietary changes.

Our results also highlight the important role that dieticians and non-medical sources (internet, books, magazines, social media) play, given that despite 90% of respondents desired dietary information, only 66% expressed interest in receiving guidance from their oncologist or hematologist. This study indicates that though patients with PCDs are inclined to eat more healthfully post-diagnosis, the majority currently do not receive this information from physicians and may benefit from professional input from dietitians or physicians to alleviate any uncertainties regarding diet and nutrition.

Strengths of this study include the large sample size. A limitation includes the flexible branching logic of the survey instrument which allowed patients to selectively answer certain questions. Thus, we captured differing response rates across some sections (i.e., Table 1 versus Table 2 questions) as participants were less likely to complete questions further along the survey. Alternatively, this scheme allowed for a larger clinical sample size. The retrospective nature of surveys may have led to recall bias in patients, producing an overestimation of effect size when comparing pre-diagnosis habits with those post-diagnosis. Although the selection of HealthTree Cure Hub as the platform to disseminate the survey lent itself to greater outreach amongst patients, this may have led to a self-selection bias from patients who are interested in this topic and may already have made dietary changes. Beyond selection bias, the generalizability of these results may be constrained by the low response rate (5.3%) given 421 responses were captured despite 8000 site visitors. However, the exact number of patients active on the site during the survey period is unknown and is likely under 8000.

## Conclusions

To summarize, our survey reveals a missed opportunity between patients’ need for dietary advice and the potential for hematologists and oncologists to provide helpful counsel. Patients with PCDs are interested in dietary advice from hematologists and oncologists to make healthful dietary switches. Most patients currently make dietary changes post-diagnosis. However, they receive advice pertaining to diet and nutrition from non-medical sources and report barriers related to lack of consistent information. Our findings highlight a need for additional research into standardized guideline (AICR and ACS) implementation as well as for the development of PCD-specific guidelines by hematologists and oncologists. Further disease focused dietary studies among patients with PCDs, especially those aiming to assess the impact of defined dietary interventions on biomarkers of disease prognosis and survival outcomes (e.g., NCT04920084), are essential to fill this gap.

## Data Availability

The datasets generated during and/or analyzed during the current study are available from the corresponding author on reasonable request.
